# Hospital-Community Interactions Foster Coexistence between Methicillin-Resistant Strains of *Staphylococcus aureus*

**DOI:** 10.1371/journal.ppat.1003134

**Published:** 2013-02-28

**Authors:** Roger Kouyos, Eili Klein, Bryan Grenfell

**Affiliations:** 1 Department of Ecology and Evolutionary Biology, Princeton University, Princeton, New Jersey, United States of America; 2 Division of Infectious Diseases and Hospital Epidemiology, University Hospital Zürich, University of Zürich, Zürich, Switzerland; 3 Center for Advanced Modeling, Department of Emergency Medicine, Johns Hopkins University, Baltimore, Maryland, United States of America; 4 Center for Disease Dynamics, Economics & Policy, Washington, D.C., United States of America; Emory University, United States of America

## Abstract

Methicillin-resistant *Staphylococcus aureus* (MRSA) is an important cause of morbidity and mortality in both hospitals and the community. Traditionally, MRSA was mainly hospital-associated (HA-MRSA), but in the past decade community-associated strains (CA-MRSA) have spread widely. CA-MRSA strains seem to have significantly lower biological costs of resistance, and hence it has been speculated that they may replace HA-MRSA strains in the hospital. Such a replacement could potentially have major consequences for public health, as there are differences in the resistance spectra of the two strains as well as possible differences in their clinical effects. Here we assess the impact of competition between HA- and CA-MRSA using epidemiological models which integrate realistic data on drug-usage frequencies, resistance profiles, contact, and age structures. By explicitly accounting for the differing antibiotic usage frequencies in the hospital and the community, we find that coexistence between the strains is a possible outcome, as selection favors CA-MRSA in the community, because of its lower cost of resistance, while it favors HA-MRSA in the hospital, because of its broader resistance spectrum. Incorporating realistic degrees of age- and treatment-structure into the model significantly increases the parameter ranges over which coexistence is possible. Thus, our results indicate that the large heterogeneities existing in human populations make coexistence between hospital- and community-associated strains of MRSA a likely outcome.

## Author Summary

One of the most notorious cases of antibiotic-resistant bacteria is methicillin-resistant *Staphylococcus aureus* (MRSA), which causes diseases ranging from skin and soft-tissue infections to pneumonia and septicemia. Traditionally, MRSA was mainly hospital-associated, but in the past decade community-associated strains have spread widely. Typically drug-resistant bacteria have lower reproduction or transmission rates, called a fitness cost. Because this cost is estimated to be significantly lower for community-associated strains, it has been predicted that these will eventually replace the hospital-associated strains. However, hospital-associated strains are resistant against a greater variety of antibiotics, which may compensate for the higher fitness cost. Here, we integrate realistic data on drug-usage, resistance profiles, contact, and age structures into a mathematical model of MRSA transmission to predict the competition between hospital- and community-associated strains. We find that for a realistic degree of population structure it is likely that both strains of MRSA will coexist in the long term. This results from significantly different hospitalization and antibiotic consumption rates between age groups. In particular, elderly individuals have much higher rates of antibiotic usage and hospitalizations than other age groups. This generates a situation where community-associated strains can predominate in the community but are outcompeted in the hospital, resulting in coexistence in the population.

## Introduction

Over the past ten years community-associated strains of methicillin-resistant *Staphylococcus aureus* (CA-MRSA) have emerged and spread rapidly, accounting for large increases in disease both in the community and in the hospital [1], [2]. While originally thought to be primarily a hospital-associated pathogen (HA-MRSA), the emergence of a community-associated strain, which has a different genetic background [2] and drug susceptibility profile [3], [4], has raised questions about how the epidemiology and the ecology of the disease will evolve, particularly with respect to which strain will predominate.

MRSA resistance is mediated by the integration of a staphylococcal cassette chromosome mec (SCC*mec*) in a site-specific manner into the staphylococcal genome [5]. Eight different SCC*mec* allotypes, as well as numerous subtypes, which encode varying levels of resistance to multiple antibiotics, have been described to date [2]. In vitro growth assays have demonstrated an inverse correlation between resistance level and growth rate [6], [7], which presumably limits the spread of HA-MRSA strains – characterized by high levels of resistance to multiple antibiotics –beyond hospitals [8], [9]. On the other hand, the maintenance of resistance in the most common community-associated strains, which are characterized by resistance to only a limited set of antibiotics, seems to cause only a negligible reduction in the growth rate relative to non-resistant strains [10], [11]. This in turn accounts for its wide dissemination in the community and the assumption that it is more easily transmitted than HA-MRSA strains [2].

Given the presumed relatively lower cost of resistance in CA-MRSA, it has been speculated that CA-MRSA strains may eventually replace HA-MRSA strains in the hospital [12], [13]. However, the high costs of resistance for HA-MRSA strains might be offset by the fact that they are resistant to a much broader range of antibiotics than CA-MRSA strains [3], [4]. If this were the case, coexistence between the two strains might be a plausible long-term scenario. In fact, a recent empirical study [14] indicates that despite a substantial increase in patients infected with CA-MRSA strains, the frequency of HA-MRSA strains in the hospital has remained remarkably stable.

Intuitively, both outcomes, coexistence and replacement, are possible. On the one hand, replacement, or competitive exclusion, is the standard outcome expected by ecological theory for two strains occupying the same ecological niche. Accordingly, explaining observed coexistence in other bacterial pathogens has proven challenging [15], [16]. On the other hand, structural differences in hospital and community populations may impose sufficiently different selective advantages to allow coexistence. Thus, HA-MRSA with its broad resistance spectrum may be better adapted to the hospital environment where antibiotic use is common, whereas CA-MRSA may be better adapted to the community, where antibiotic use is less frequent and hence a broad resistance spectrum cannot compensate for the reduced transmissibility of HA-MRSA. One factor, which may counteract this mechanism, is that the average length of stay of a patient in the hospital (4–5 days) is much shorter than the time until a patient colonized with MRSA clears the bacteria (several 100 days [17]). Accordingly, a given colonization (bacterial population within a host) almost never experiences only the hospital environment, hence making local adaptation difficult. It follows from the above reasoning that addressing the question of coexistence is not possible from the hospital perspective alone, but instead it is necessary to take both hospital and community populations (and their frequent exchange) into account.

While this question of coexistence is an interesting ecological problem, it is also an important question for public health as the outcome of the interaction between CA-MRSA and HA-MRSA may have epidemiological and clinical consequences: HA-MRSA has a much broader resistance spectrum than CA-MRSA [18] and may therefore be more difficult to treat. Moreover, the two strains also differ with respect to their pathogenicity. While CA-MRSA has primarily been associated with skin- and soft-tissue infections, there have also been suggestions that it may be more invasive and virulent than HA-MRSA [2]. If CA-MRSA completely replaces HA-MRSA, hospitals might be confronted with a more virulent but also more treatable pathogen. Moreover, their ability to replicate and transmit in the community may mean significantly more infections as well. In this analysis, we use epidemiological models which integrate realistic data on drug-usage frequencies, resistance profiles, contact, and age structures to assess the impact of competition between HA- and CA-MRSA strains. In particular, we examine the likelihood of coexistence as an outcome.

## Methods

### Models

We considered three epidemiological models of increasing complexity and assessed how the interaction between hospital and community populations could lead to stable coexistence between HA- and CA-MRSA. The basic model assumes that all individuals, regardless of age, have similar hospital admission and discharge rates as well as antibiotic usage rates. In this case the only difference between the hospital and the community is the usage frequency of antibiotics, which may lead to selection favoring one strain in the community and the other in the hospital. Next we considered two extensions of this model. First we examined how heterogeneity between age-classes with respect to hospitalization rates and antibiotic usage impacted coexistence between strains in each setting. Second, we explicitly distinguished between treated and untreated patients, thereby capturing the prophylactic effect of treatment, which is likely to be much stronger in the hospital than in the community.

The epidemiological dynamics of CA- and HA-MRSA were described by set of ordinary differential equations. Based on estimates from the literature and an analysis of public-use data in the United States, we assume differing dynamics of colonization, infection, and antibiotic use between the hospital and the community and consider how differing implementations of the host population structure impacts the dynamics of each strain and examine the parameter ranges over which coexistence occurs. In each of these models, the possibility of coexistence between HA- and CA-MRSA for a given parameter-combination was determined by an invasibility analysis: First, the system is allowed to reach the equilibrium with one strain only (burn-in time: 5×10^4^ days); then the other strain is introduced at low abundance; if the introduced strain increases in frequency after the introduction, we say that it can invade the equilibrium determined by the resident strain. If both strains (HA-MRSA and CA-MRSA) can invade the equilibrium of the other strain, this indicates coexistence.

### Basic model

In the basic model we assume that populations in the hospital and community are homogenous. The two populations are connected through admission into the hospital population (with rate *a*) and discharge into the community with rate (*d*) and are each subdivided into individuals that are uncolonized (*S_C_* and *S_H_*), colonized with CA-MRSA (*C_C,CA_* and *C_H,CA_*), and colonized with HA-MRSA (*C_C,CA_* and *C_H,CA_*). In the community, colonized individuals infect uncolonized individuals with a transmission rate *β_C,CA_* for CA-MRSA and *β_C,HA_* for HA-MRSA. In the hospital the corresponding rates are *β_H,CA_* and *β_H,HA_*. We assume that regardless of location, in the absence of treatment HA-MRSA suffers a fitness cost (*s*) against CA-MRSA, which is assumed to be due to a reduced transmission rate; i.e. *β_X,HA_ = β_X,CA_*(1*-s*). Colonization can be cleared either spontaneously, with rate *c_BL_*, or through treatment. Since colonization is significantly more common than infection, we assume that antibiotic use is independent of the colonization status of the patient. Antibiotic use occurs at rate *τ_C_* and *τ_H_* in the hospital and community, respectively. As treatment is in most cases not specific for *S. aureus,* we assume that the probability (*f_C,X_* and *f_H,X_*) that a given course of treatment is effective against either strain corresponds to the proportion of drugs to which each is susceptible among all prescribed drugs (see parameters section). We further assume that even if the drug consumed is effective, there are reasons other than antibiotic resistance that a bacterial colonization may not be cleared, i.e. effectively treated patients clear the bacterial population only with a probability *c_T_<1*. The basic model is described by the following set of ordinary differential equations:
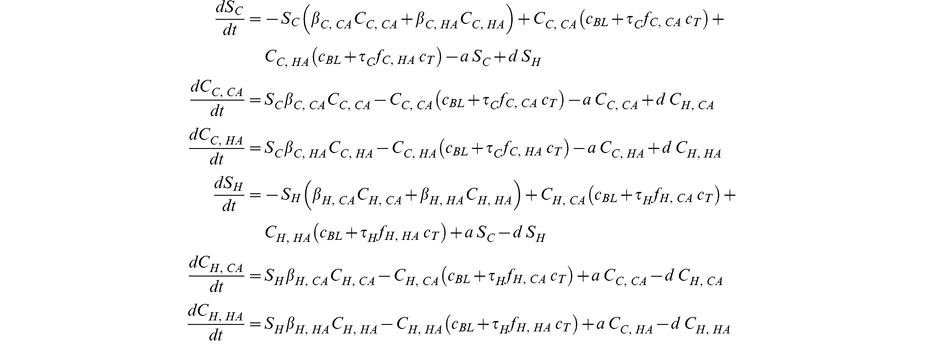



### Age-structured model

**Figure 1:**
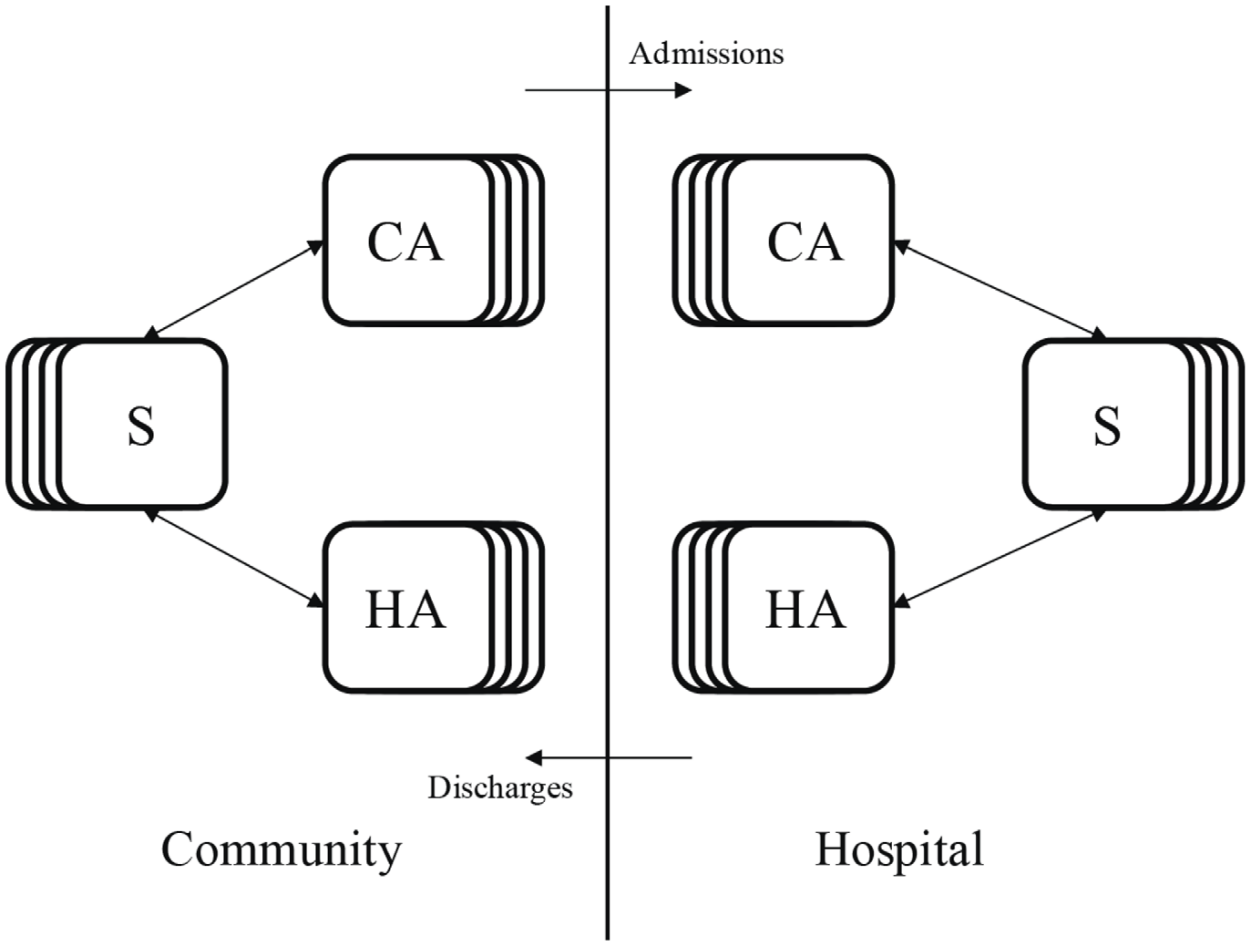
Flow diagram of the age structured model. Susceptible individuals (S), which are structured into multiple age classes, can be colonized by CA- or HA-MRSA in the community or in the hospital. Colonized individuals, which are also structured by age, clear the pathogen either by treatment or through natural immune clearance. Individuals move between the hospital and the community at the same rate regardless of colonization status.

The age-structured model is derived from the basic model by sub-dividing each compartment into 18 different age classes (five-year bins for the ages from 0 to 85 and one bin for 85+). For instance, the compartment *S_C_* (uninfected individuals in the community) is subdivided into the compartments *S_C_^1^, S_C_^2^… S_C_^18^*, where *S_C_^1^* covers susceptible individuals of age 0–5, *S_C_^2^* of 5–10, etc. Admission rates, *a_j_*, and discharge, *d_j_*, rates are assumed to depend on the age class *j* (see parameters section). The impact of age structure on contact rates is captured by assuming that the transmission rates in the community, *β_C,X_^j,k^*, are proportional to the frequency of physical contacts (divided by the number of people in that age class). Because detailed contact data from the US were not available, we used data on contact rates measured in the UK [19]. Finally, the treatment rate *τ_C_^j^* in the community also varies with age class *j*. Similar data on the age-dependency of contact rates and treatment rates in the hospital were not available to our knowledge. Because contact rates are likely to be more uniform in the hospital, since most of transmission is indirect, we made the conservative assumption that contact and treatment rates in the hospital are uniform regardless of age. The age-structured model is described by the following set of ordinary differential equations:
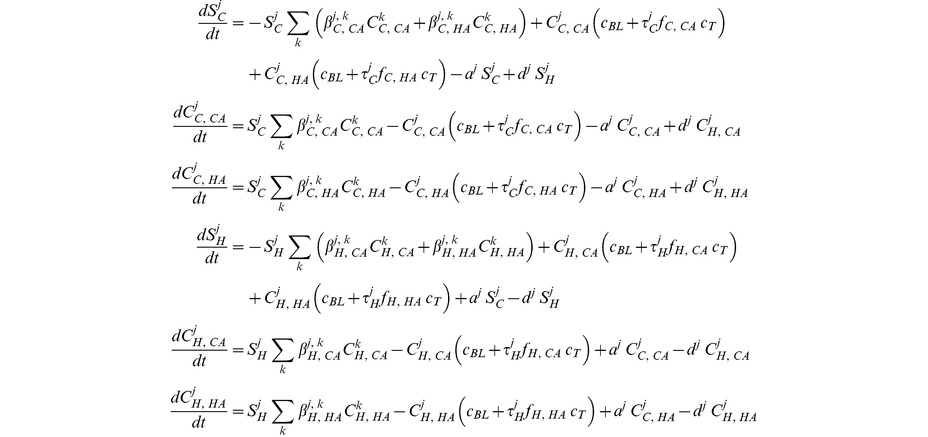
A diagram of the age-structured model is found in Figure 1.

### Treatment-structured model

The treatment-structured model (Equation S1 in the online supplementary material) is derived from the basic model by subdividing each compartment according to treatment status. Specifically, we distinguish between four treatment classes: 1) Untreated (*U*); 2) treated with a drug that is effective against neither CA-MRSA nor HA-MRSA (treatment *T*(1)); 3) treated with a drug that is effective against CA-MRSA but not HA-MRSA (treatment *T*(2)); and 4) treated with a drug that is effective against both CA-MRSA and HA-MRSA (treatment *T*(3)). Treatment with a drug from class *j* is initiated at rate *τ_C_^j^* and *τ_H_^j^*. Upon treatment initiation with an effective drug, the infection is either cleared immediately with a probability *c_T_* or alternatively remains colonized (this is an approximation to the real dynamics in which the patient would clear after a given amount of time). Finally, treated individuals stop treatment at a rate *ρ* (i.e. the inverse duration of antibiotic use). A diagram of the model is found in Figure S1.

The treatment-and-age-structured model is derived from the treatment-structured model in the same way the age-structured model is derived from the basic model.

### Parameters

**Figure 2:**
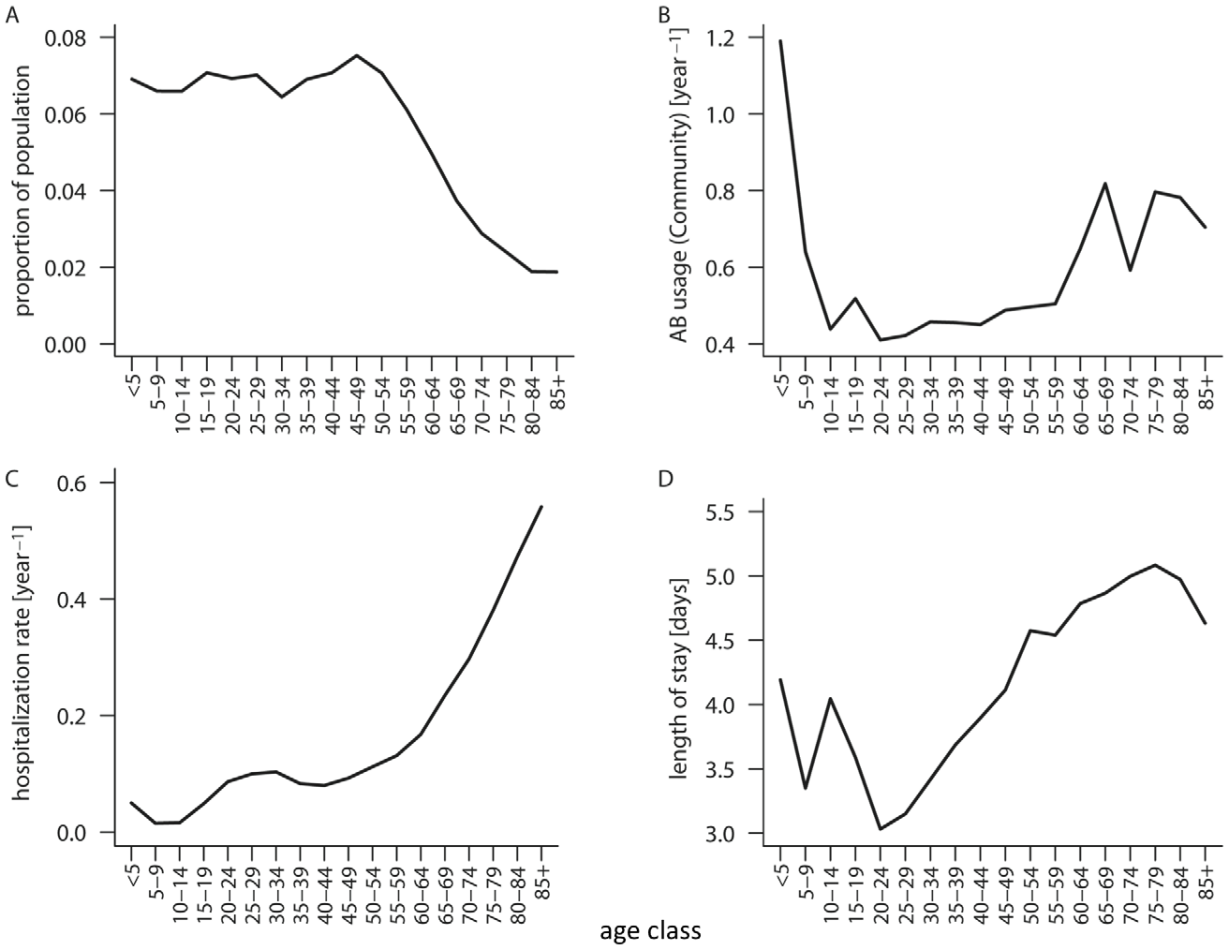
Distribution of the US population over age classes, age dependency of hospitalizations, treatment rates, and durations of hospitalization in the US. Data are shown for the 18 age classes used in the age-structured model.

Our models integrate realistic values for drug-usage frequencies, resistance profiles, age structure, age-dependent contact patterns, and hospitalization rates. Usage frequencies, age distribution, hospitalization rates, and the mean length of stay in the hospital were estimated from publicly available data by five-year age groups. Figure 2 summarizes the age-dependency of population size, hospitalization rates, length of stay in the hospital, and antibiotic usage rates in the community. As data on the age-dependency of treatment and contact rates are available for the community only, we make the conservative assumption that these rates are homogenous in the hospital (see discussion). In contrast to these parameters there is large uncertainty concerning the magnitude of transmission rates (particularly in the hospital) and especially concerning the degree to which the transmission rate of HA-MRSA is reduced compared to that of CA-MRSA (i.e. the selective cost of HA-MRSA). Therefore we vary these parameters over broad ranges. A summary of the parameter values and ranges can be found in Table 1.

**Table 1:** Default model parameters of the basic model.

Parameter	Explanation	Default Value
*a*	Admission rate to hospital	0.00032 d^−1^ *
*d*	Discharge rate from hospital	0.25 d^−1^ *
*τ_C_*	Treatment rate in community	0.0015*d^−1^* **
*τ_H_*	Treatment rate in hospital	0.2 *d^−1^* ***
*f_H,HA_*	Probability that a treated HA-MRSA colonized individual receives effective drugs in the hospital	0.1#
*f_H,CA_*	Probability that a treated CA-MRSA colonized individual receives effective drugs in the hospital	0.9#
*f_C,HA_*	Probability that a treated HA-MRSA colonized individual receives effective drugs in the community	0.03#
*f_C,CA_*	Probability that a treated CA-MRSA colonized individual receives effective drugs in the community	0.7#
*c_T_*	Probability that patient clears infection given appropriate treatment	0.5 [43]
*c_BL_*	Base-line clearance rate	1/300 d^−1^ [44]
*R_0_^CA,C^*	Basic reproductive ration of CA-MRSA in community	1.4##
*R_A_^HA,H^*	Single-admission basic reproductive number of HA-MRSA in hospital	Variable (0.5–1.5)###
*s*	Reduction of transmissibility of HA-MRSA (*β_X,HA_ = β_X,CA_*(1*-s*))	Variable (0.1–0.5)
*β_X,Y_*	Transmission rate of strain x in setting y	Determined by R_0_ and s

* Average transmission and discharge rate in the US (see methods section); for age-class dependent rates see Figure 1.

** Average number of antibiotic prescriptions in the US (data from the data from the National Ambulatory Medical Care Survey (NAMCS) and the National Hospital Ambulatory Medical Care Survey (NHAMCS)); see Table S1.

*** Polk et al [20] measured antibiotic use in 130 US hospitals. They found that 60% of all discharged patients received at least one dose of an antibacterial drug. With an average length of stay of 4 days (see *) a daily treatment rate of 0.2 leads to 0.55% of patients receiving treatment during their stay.

# *f_X,Y_* was calculated as the fraction of drugs used in setting X that are effective against the strain Y (see methods section).

## The basic reproductive ratio of CA-MRSA in community was determined under the assumption that this strain pays no cost of resistance; i.e. it has the same transmission rate as methicillin-sensitive *S aureus*. The latter colonizes about 30% of individuals in the community and with an R_0_ of 1.4 the expected prevalence is 1-1/1.4 = 0.29.

### The single-admission basic reproductive number of HA-MRSA in the hospital corresponds to the number of secondary infections caused by a single colonized individual admitted to a hospital containing only susceptible individuals and is given by the dominant eigenvalue of the next-generation matrix B V^−1^. The matrix B is given by B_ij_ = *β_HA,H_^ij^ S^i^,* [with *S^i^* the frequency of susceptibles of age-class *i* in the disease-free equilibrium in the hospital. In the absence of age structure *S^i^ = a/d for all i*. In the presence of age structure S^i^ = a_i_/d_i_ *(the proportion of the total population in age group *i*) (see Figure 2)]. The matrix V is given by V_ij_ = *d_j_+c_BL_+τ_H_ c_T_ f_H,HA_* for *i = j* and V_ij_ = 0 for *i≠j*.

The number of hospitalizations and average length of stay for each age group was estimated from the Nationwide Inpatient Sample (NIS), Healthcare Cost and Utilization Project, Agency for Healthcare Research and Quality for the year 2008. The NIS contains data on ∼8 million records of hospital stays annually from about 1,000 hospitals, approximating a 20% stratified sample of US community hospitals, and includes all nonfederal, short-term, general, and specialty hospitals, such as obstetrics-gynecology, ear-nose-throat, orthopedic, and pediatric institutions. The NIS includes public hospitals and academic medical centers but excludes long- and short-term acute rehabilitation facilities, psychiatric hospitals, and alcoholism and chemical dependency treatment facilities. Hospitalization rates were calculated as the average number of hospitalizations per-person per-day by age group. The numbers of individuals for each age group were obtained from the US Census bureau's annual estimates of the resident population by five-year age groups (www.census.gov).

Antibiotic usage in the community was estimated based on data from the National Ambulatory Medical Care Survey (NAMCS) and the National Hospital Ambulatory Medical Care Survey (NHAMCS) for 2008. NAMCS is an annual national survey of visits to non-federally employed office-based physicians who are primarily engaged in direct patient care, and NHAMCS is designed to collect data on the utilization and provision of ambulatory care services in hospital emergency and outpatient departments and in ambulatory surgery centers. Weighted patient level data was used to estimate the annual number of prescriptions for antibiotics that were written for each age group. The usage rate was calculated as the average number of prescriptions written per person per day per age group. Antibiotic usage in the hospital was estimated based on the data from [20], which reported antibiotic use from 130 US hospitals (see Table 1).

To calculate the approximate effectiveness of community antibiotic usage on CA- and HA-MRSA, we calculated the number of prescriptions for each antibiotic class and, based on assumptions about the effectiveness of each antibiotic against CA- and HA-MRSA, we estimated the percentage of drug usage that was effective against each pathogen (Supplementary Table S1). The effectiveness of drugs used in the hospital is similar to the community though skewed towards some of the more effective drugs [20], [21]. Thus, we assume that the effectiveness of antibiotic usage on CA- and HA-MRSA is slightly more effective in the hospital than in the community (see Table 1).

## Results

In order to explore the possibility of coexistence between HA-MRSA and CA-MRSA, we consider a series of epidemiological models of increasing complexity. The simplest, basic model ignores all population structure beyond the distinction between hospital and community. We then extend this basic model by incorporating age- and treatment-heterogeneities in accordance with published data (see Methods).

**Figure 3:**
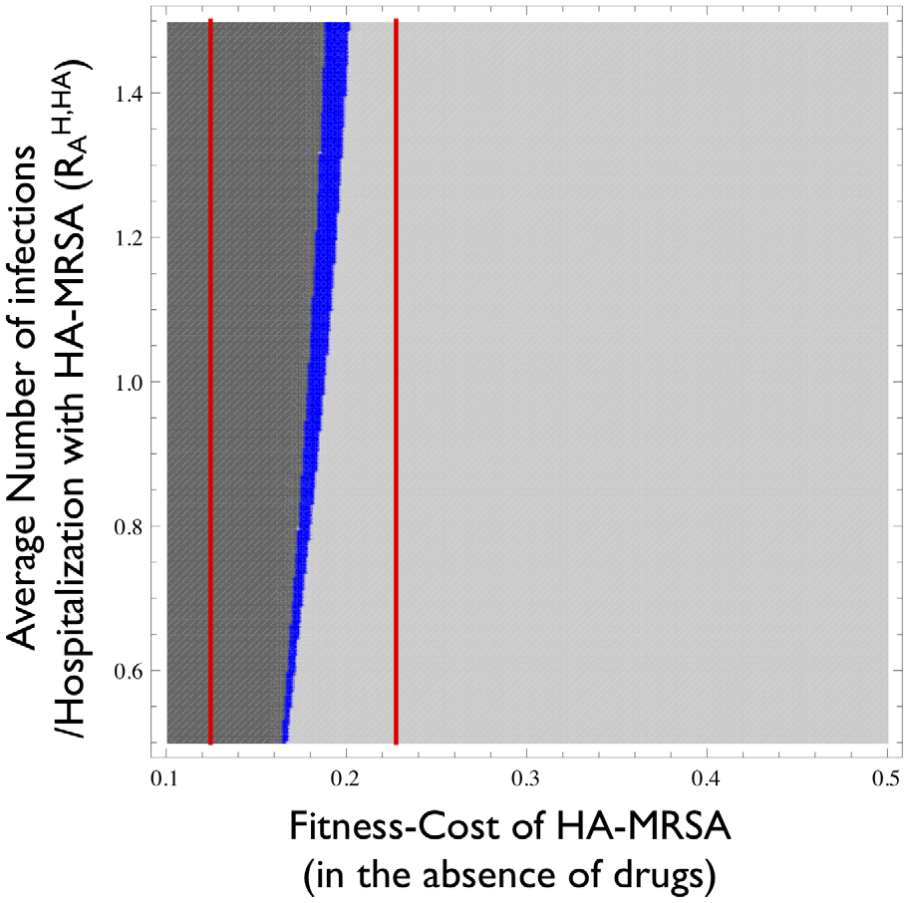
Parameter range for which HA-MRSA and CA-MRSA coexist. The blue area indicates the parameter-combinations for which HA-MRSA and CA-MRSA coexist. The dark grey region indicates the parameter-combinations in which HA-MRSA cannot be invaded by CA-MRSA. The light-grey region indicates parameter-combinations in which CA-MRSA cannot be invaded by HA-MRSA. The range between the two red lines corresponds to fitness costs for which selection in the hospital and community act in opposite directions (i.e. CA-MRSA is fitter in the community and HA-MRSA is fitter in the hospital). The x-axis corresponds to the fitness disadvantage of HA-MRSA compared to CA-MRSA in the absence of effective therapy. The y-axis corresponds to the average number of secondary infections caused by a single colonized individual admitted to a hospital containing only susceptible individuals (single-admission reproduction number *R_0_^HA,H^* [41], see Table 1).

For the basic model, which ignores both age- and treatment-structure, we find that the interaction between the hospital and the community can in principle generate coexistence between HA- and CA-MRSA. However, we observed this outcome only for a relatively narrow band of fitness-costs for HA-MRSA (Figure 3). Moreover, the width of this band decreases with decreasing transmissibility in the hospital, which we quantified as the average number of secondary cases caused by the admission of one patient to the hospital containing only susceptible patients (y-axis in Figure 3). Of note, the parameter range for which coexistence occurs is much narrower than the parameter range for which HA-MRSA is fitter than CA-MRSA in the hospital but less fit than CA-MRSA in the community (see red bars in Figure 3). Thus opposite directions of selection are not a sufficient condition for coexistence. This is due to the significant linkage between the two systems caused by the high-turnover (admission/discharge) in the hospital, which results in the two strains being cycled into and out of the community, making it harder for the two strains to coexist. If the cost of HA-MRSA exceeds the values in the coexistence range, the equilibrium in which only CA-MRSA is present becomes stable; if the cost is smaller, the equilibrium in which HA-MRSA can exclude CA-MRSA becomes stable. Such a narrow range of coexistence is not unexpected for a homogeneous model. However, the epidemiology of MRSA exhibits several heterogeneities which can stabilize coexistence over a broader range of conditions.

### Heterogeneities increase the range of coexistence

**Figure 4:**
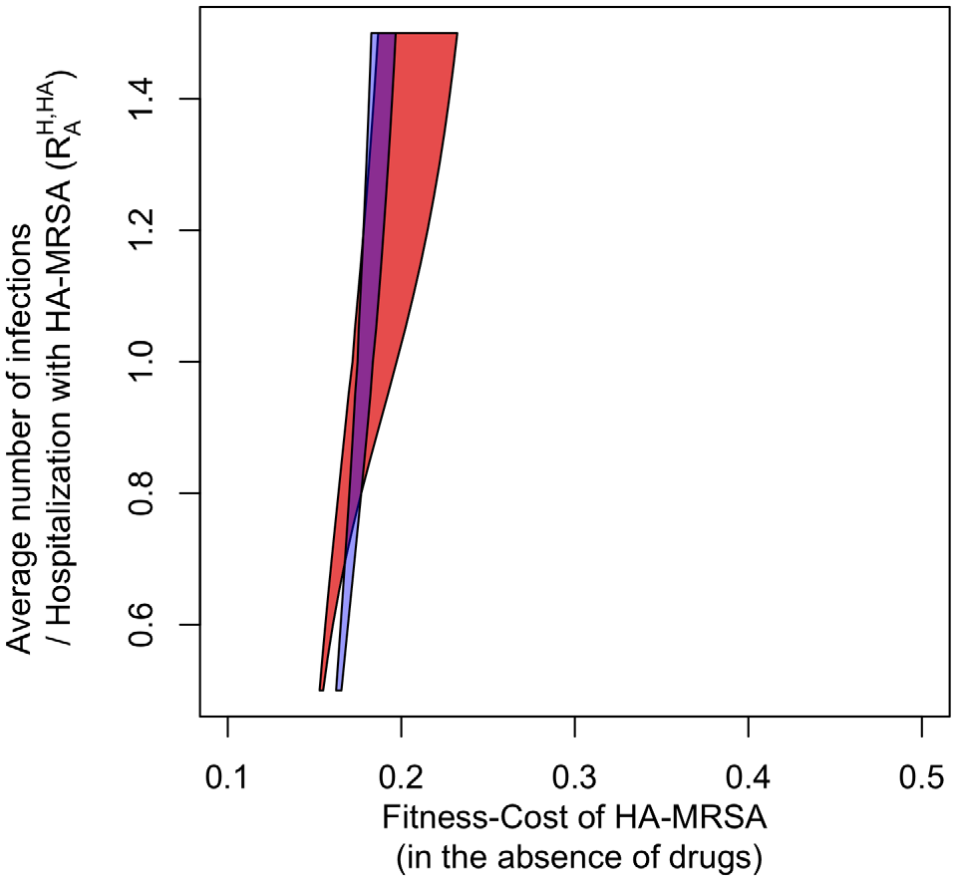
The blue area indicates the parameter combinations for which HA-MRSA and CA-MRSA coexist in the basic model. The red area indicates co-existence in the age-structured model. Axes and parameter values are the same as in Figure 3. Note that in order to assure comparable transmissibility measures as y-axes in the homogeneous and heterogeneous models, we measure transmissibility as the single-admission reproductive number *R_0_^HA,H^* – i.e. the dominant eigenvalue of the next-generation matrix of the hospital [41], [42] (see Table 1).

We included age-dependent transmission rates for the community by assuming that transmission rates are proportional to the rate of physical contact [19]. Including age structure in this manner substantially broadens the parameter range over which HA- and CA-MRSA can coexist (Figure 4). This increase is due in part to relative differences in hospitalization between younger and older individuals, which changes the relative difference in selection between HA- and CA-MRSA strains. Because the hospital admission rate and the average length of stay increases as individuals age, older individuals are more likely to spend time in the hospital, where MRSA is favored, and consequently they are more likely to be colonized with HA-MRSA which increases the range over which HA-MRSA is able to persist despite an influx of CA-MRSA from the community. Moreover, the number of physical contacts an older person has in the community is considerably lower than the corresponding number for young persons. This in turn further reduces the selective advantage of CA-MRSA in old age classes. As physical contact occurs preferentially between members of the same or neighboring age classes [19], this further contributes to maintaining the association between age and strain.

**Figure 5:**
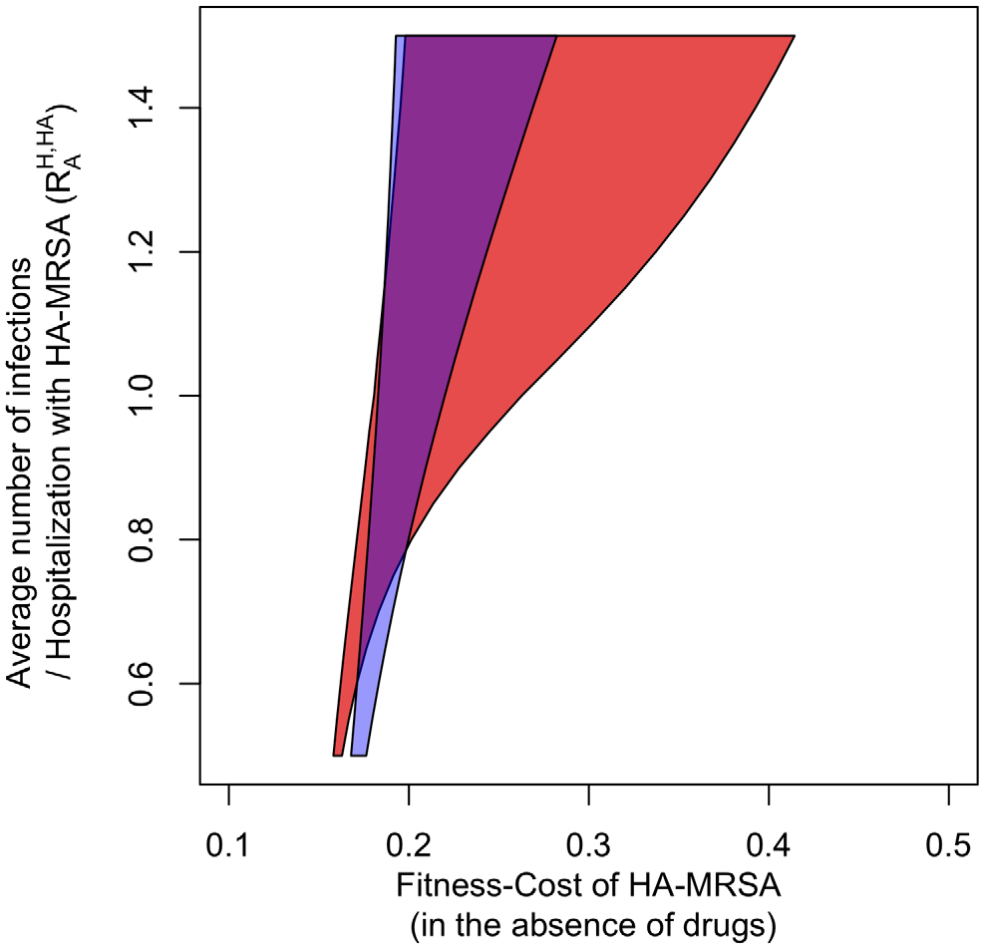
The blue area indicates the parameter combinations for which HA-MRSA and CA-MRSA coexist in the treatment-structured model. The red area indicates coexistence in the treatment- and age-structured model. Axes and parameter values are the same as in Figure 3.

An additional source of heterogeneity is treatment itself. We take this heterogeneity into account by explicitly tracking the treatment status of patients and assuming that individuals receiving a given antibiotic cannot be colonized with strains that are susceptible to this drug. Including treatment heterogeneity in this way leads to an additional, substantial extension of the parameter range over which coexistence occurs (Figure 5). This is because antibiotic prophylaxis of colonization creates a substantial additional selective advantage for HA-MRSA (which has the broader resistance spectrum) in the hospital. However, whereas the fraction of protected patients is large in the hospital, it is negligible in the community and hence prophylaxis does not substantially increase the fitness of HA-MRSA in the community. Treatment heterogeneity and age-structure act synergistically to increase coexistence, such that the broadest coexistence range can be observed for the treatment- and age-structured model (see Figure 5).

### Different costs of resistance in hospital and community

**Figure 6:**
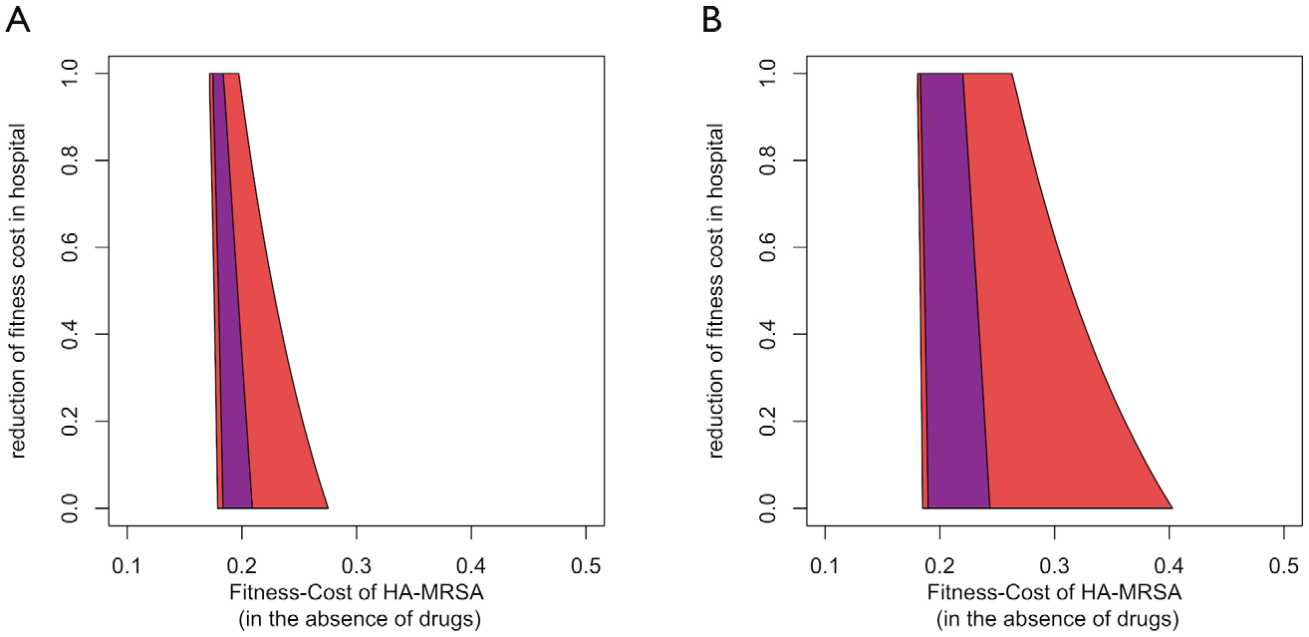
A) The blue area indicates the parameter combinations for which HA-MRSA and CA-MRSA coexist in the basic model. The red area indicates coexistence in the age-structured model. **B**) The blue area indicates the parameter combinations for which HA-MRSA and CA-MRSA coexist in the treatment-structured model. The red area indicates co-existence in the treatment- and age-structured model. The x-axis corresponds to the fitness disadvantage of HA-MRSA compared to CA-MRSA in the community in the absence of effective therapy. The y-axis corresponds to ratio between the fitness costs of HA-MRSA in hospital and community.

HA-MRSA is most likely adapted to the hospital environment in other ways than by its broad antibiotic resistance spectrum (e.g. tolerance to disinfectants, smaller requirements of invasibility, etc.) [2], [22]. Accordingly, it is likely better able to compete against CA-MRSA (in the absence of therapy) in the hospital as opposed to the community. Taking this effect into account, we find that as the fitness-cost of HA-MRSA in the hospital decreases relative to CA-MRSA, the maximal fitness cost of HA-MRSA for which coexistence occurs is strongly increased. By contrast the minimal cost for coexistence changes only weakly, because reducing the cost of resistance in the hospital does not affect relative fitness in the community (Figure 6). Thus, context specific fitness costs further facilitate coexistence between HA- and CA-MRSA. It is remarkable that decreasing the cost of HA-MRSA in the hospital has a much stronger effect in the presence of age structure than in its absence (which indicates that age structure helps separate the hospital from the community). Thus there is a synergistic effect between age-structure and hospital specific reduction of fitness costs.

### Simulation of transient strain dynamics

The above analysis was based on the ability of one strain to invade the equilibrium defined by the presence of the other strain. This method indicates where the two strains can coexist at equilibrium and therefore allows one to assess the main ecological forces underlying coexistence and competitive exclusion. However, it has three disadvantages: First, the equilibrium might be attained only very slowly: for instance two strains might coexist for a transient period which can extend over decades even though the equilibrium analysis indicates that one strain should exclude the other. Second, even if the two strains coexist one of them might attain only very low levels (i.e. even though the two strains can coexist in theory, almost all infections are caused by one single strain). Third, the pairwise-invasibility approach only allows an analysis of the competitive interaction of two strains, whereas, in reality, several *S. aureus* strains compete with each other: Notably, HA-MRSA and CA-MRSA compete with methicillin sensitive *S. aureus* (MSSA), which could modify their interaction.

In order to address these issues, we considered a more pragmatic definition of coexistence: We initiated the population either with HA-MRSA as the only resident strain or with two resident strains (HA-MRSA and MSSA) and ran the simulation for 30 years. Then we add the new strain (CA-MRSA) and examined how the frequencies of each changed over time. Specifically, we tested after 10, 20, 50, 100, and 200 years, which strains still exist in substantial frequency (using a threshold of 5%). Note that we focused here only on the invasion of HA-MRSA/MSSA equilibrium by CA-MRSA rather than the opposite, since the former describes the current epidemic development (whereas the latter is merely of theoretical interest).

**Figure 7:**
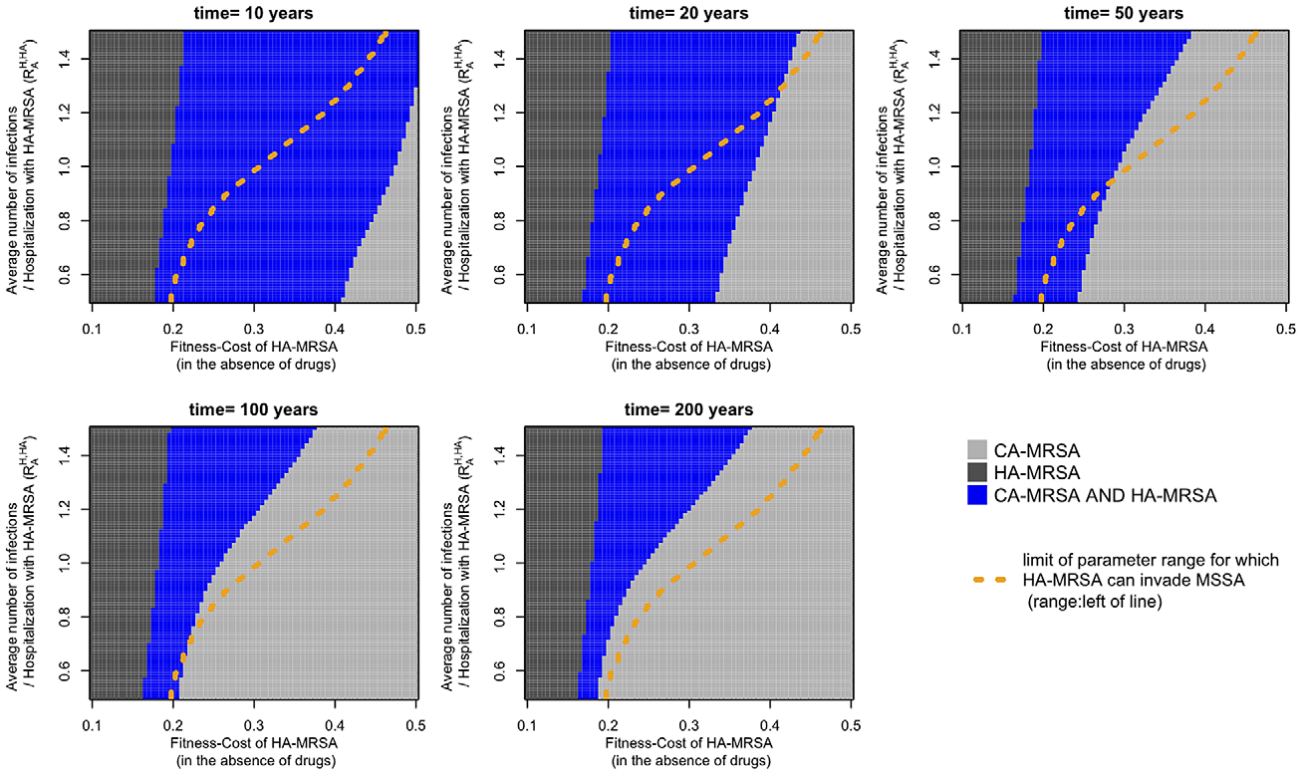
Coexistence between HA-MRSA and CA-MRSA in the transient phase after the introduction of CA-MRSA into the HA-MRSA-infected host population in the treatment- and age-structured model (corresponding to the red area in Figure 5). Colors indicate which strains have frequencies >5% among the colonized patients in the hospital (HA-MRSA) and the community (CA-MRSA): Blue indicates coexistence (i.e. both strains >5%), dark grey indicates HA-MRSA only, and light grey CA-MRSA only. The dashed orange line delimits the parameter region in which HA-MRSA can invade MSSA (criterion for invasion: frequency of MRSA >5%, 50 years after its introduction; see Figure S2).

We first considered the interaction between CA-MRSA and HA-MRSA (in analogy to the above analyses). We find that the two strains can coexist during a long transient phase (10–50 years) for a broad range of conditions, which do not support coexistence at equilibrium (see Figure 7). Moreover, we can substantially reduce the range of realistic parameters by considering the interaction between HA-MRSA and MSSA: as we know that HA-MRSA has attained substantial frequencies (in the USA at least) after <50 years of methicillin use, the model is only consistent with reality for those parameter-combinations for which this is the case. Figure S2 shows that an invasion of HA-MRSA into the MSSA equilibrium is only possible if the fitness cost of HA-MRSA is below a threshold that is dependent upon the average number of secondary cases caused by the admission of one patient to the hospital containing only susceptible patients (*R_0_^HA,H^*). This threshold is indicated in Figure 7 by the dashed orange line. As it is also a fact that CA-MRSA was able to invade HA-MRSA, the realistic parameter range in Figure 7 is delimited by the dark grey area (corresponding to parameter values where the CA-MRSA invasion is impossible) to the left and the orange line to the right. Thus, Figure 7 indicates that we would expect a long-term coexistence between HA-MRSA and CA-MRSA for most realistic parameter combinations.

**Figure 8:**
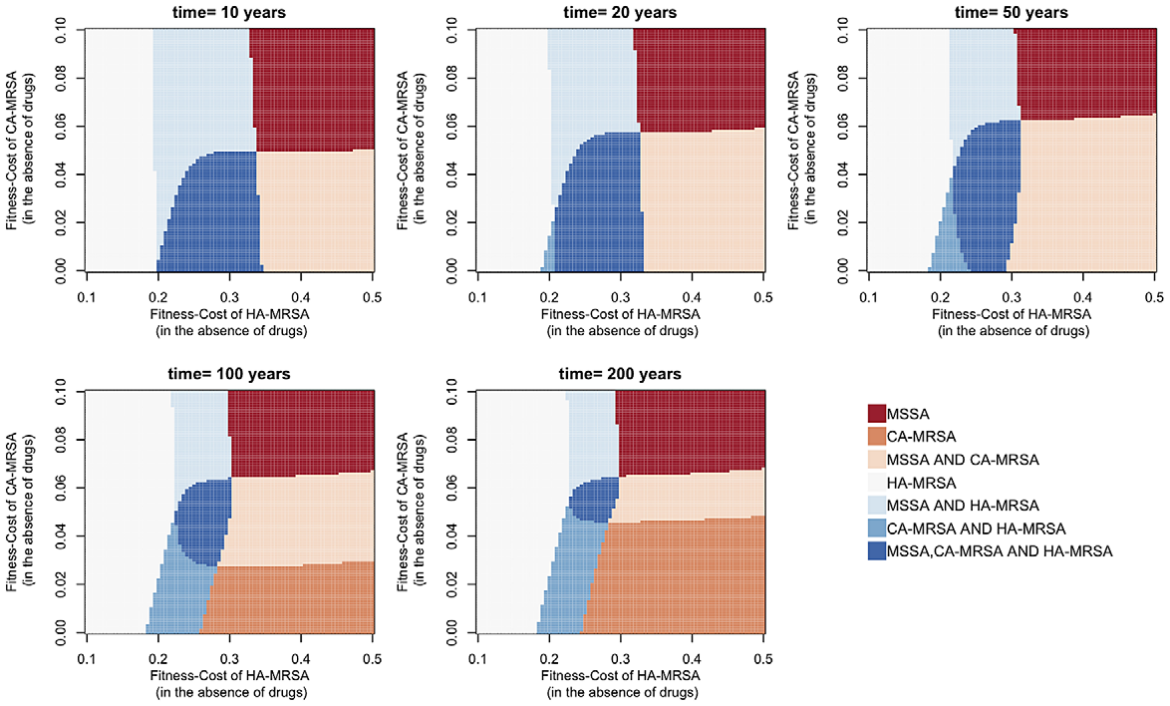
Coexistence between MSSA, HA-MRSA and CA-MRSA in the transient phase after the introduction of CA-MRSA into the HA-MRSA/MSSA-infected host population in the treatment- and age-structured model. Colors (see legend) indicate which strains have frequencies >5% among the colonized patients in the hospital (HA-MRSA) and the community (MSSA/CA-MRSA).

When we consider the interaction between all three strains by including MSSA as one of the initial resident strains, we find that the parameter range in which all three strains can coexist shrinks successively with increasing time (see Figure 8) and eventually vanishes (results not shown). This is not unexpected, as the model structure assumes that the hospital and the community are two different ecological niches, which can thus maximally support the coexistence of only two strains over the long-term. However, we do find that all three strains can coexist for a broad range of conditions during a long transient time-span of several decades. Overall, these results indicate that transient effects can strongly extend the range of coexistence, and even allow for long-term de-facto coexistence where this would not be expected at equilibrium.

## Discussion

We examined how differences in age-structured patterns of antibiotic use and hospitalization rates can promote coexistence of CA- and HA-MRSA. Overall, our results show that hospital and community-associated strains of MRSA can coexist if the broader resistance spectrum of the hospital-associated strains is balanced by intermediate fitness-disadvantages in the absence of treatment. For such intermediate fitness costs, the hospital-associated strains have higher fitness in the hospital, where treatment rates are high, whereas community-associated strains have a higher fitness in the community were treatment rates are low. Despite opposite directions of selection, both strains are present in both environments if there is coexistence at all (see Figure S3 for example runs). This occurs because of the high rates of discharge and hospitalization, which cycle individuals between the hospital and the community. Moreover, our results also indicate that opposite directions of selection are not sufficient for maintaining coexistence. This is especially true for our basic model describing well-mixed populations in the hospital and community, in which we found coexistence only for a very narrow range of HA-MRSA fitness-costs.

Including heterogeneity in the form of realistic age- and treatment-structures into the model significantly increases the range of parameters over which coexistence can occur, making it a likely outcome. Furthermore, the fitness cost of HA-MRSA in the absence of treatment is presumably weaker in the hospital than in the community because of factors such as easier invasion due to open wounds, catheters, etc., as well as increased use of antiseptics to which the hospital strain might be better adapted. Taking this possibility into account leads to an additional, substantial increase in the range over which coexistence is likely. Thus, coexistence between HA-MRSA and CA-MRSA is a likely outcome due to the combined effect of hospital-community interactions, age-structure, treatment-structure, and possibly setting dependent fitness costs in the absence of treatment.

Coexistence is mainly dependent upon the cost of HA-MRSA being neither too high nor too low. It should be noted, however, that the upper bound for resistance costs is, in this context, more informative than the lower bound. For costs of HA-MRSA below the lower bound, we would expect that CA-MRSA could not invade the HA-MRSA equilibrium. However, such an invasion is exactly what occurred during the 1990s. Thus, we know that fitness-costs of HA-MRSA are high enough to allow the invasion of CA-MRSA. The crucial question is whether they are low enough for this invasion to stop before CA-MRSA has completely replaced HA-MRSA.

The width of the coexistence range depends strongly on how effectively MRSA can transmit in the hospital. In our simulations we quantified this transmissibility as the average number of secondary cases caused by the admission of one patient to the hospital containing only susceptible patients (R_A_). If this value is considerably smaller than one (i.e. hospitals cannot maintain the spread of MRSA on their own), then the coexistence range becomes very narrow. This is because coexistence relies on opposite directions of selection in the hospital and community environment. If however, one of these environments does contribute only very weakly to transmission, this balancing effect cannot take place. The only published estimate for R_A_ we are aware of found values of 0.68 (0.47–0.95) and 0.93 (0.71–1.21) for two Dutch hospitals; one implies a broad and one a narrow coexistence range (The same study also reported an R_A_ value of 0.16, which however corresponded to an animal derived strain) [23]. Because the Netherlands has been exceptionally successful in reducing nosocomial spread of MRSA [23], [24], R_A_ values are likely to be higher (and hence coexistence ranges broader) in most other settings. The sensitivity on R_A_ also implies that in regions with better infection control in hospitals (and hence lower R_A_) one would expect CA-MRSA to completely replace HA-MRSA and hence also to cause most MRSA infections in hospitals.

Even though our model realistically includes several levels of population structure, our analysis might still underestimate the range over which CA-MRSA and HA-MRSA can coexist. First, other types of heterogeneity might promote coexistence in a similar way as the ones discussed here. Examples include spatial heterogeneities like rural vs. urban areas, small vs. large hospitals (which would impose different levels of stochastic effects and thereby affect strain abundances [25]), the cycling of older patients into long-term care facilities [26], or the highly variable length of time individuals remain colonized [27]. We also neglected (due to the absence of data) age- or department-structured antibiotic usage rates in hospitals, though this could further promote coexistence. Temporal heterogeneity, such as the seasonal use of antibiotics might be an additional factor contributing to coexistence in MRSA [28]. We have also broadly categorized the multitude of different MRSA strains as either CA- or HA-MRSA. This diversity could also contribute to coexistence, as different strains may have different resistance phenotypes (It should be noted however that explaining the coexistence of such individual strains is an additional challenge). In addition to such heterogeneities, coexistence might be facilitated by co-infection with different strains [29], [30], either through co-colonization of the nares [30] or specialization of different strains to different anatomical sites. For instance, CA-MRSA primarily causes infections of the skin, whereas HA-MRSA infections are generally more invasive [2], [14]. However, it is not clear to what extent different MRSA strains can co-infect a host, and it has also been shown in a different context that co-infection leads only under very specific conditions to coexistence [15], [16]. Moreover, other studies have shown that colonization with MSSA can be protective from MRSA [31], [32], suggesting that competition may limit the extent of co-colonization with different strains.

Even though our model can explain the coexistence between HA-MRSA and CA-MRSA, we did not find any parameter combination that supports coexistence at equilibrium between more than two strains (HA-MRSA, CA-MRSA and MSSA). This suggests that the system as described by our model corresponds to only two ecological niches. This implies that the maintenance of the diversity within HA-MRSA and CA-MRSA has to be explained by mechanisms not included in our model (such as the geographical and temporal variation mentioned above). Moreover, Figures 7, 8, and S3 also indicate that the system approaches equilibrium only very slowly, such that a long transient maintenance of this diversity is conceivable even if it would not persist in an equilibrium state.

Our model also describes a static situation in which the properties of the strains and the age structure do not change over time. However, both demographic change in the human population and evolutionary change of the MRSA strains are likely to occur and their impact on coexistence between competing strains is an interesting question for future studies. Demographic change will most likely increase the proportion of old people in the US and most western countries. In the context of our model this means that selection will tend to favor hospital adapted strains, as the hospitalization rates are considerably higher for the old age classes. However, the direction of evolutionary change depends very strongly on the physiological constraints underlying antibiotic resistance. For instance, if CA-MRSA can increase its resistance spectrum while maintaining a high transmissibility, it could eventually out-compete HA-MRSA. If on the other hand a higher fitness cost is the inevitable consequence of a broad resistance spectrum, then such a replacement is unlikely to occur. Such evolutionary changes may be particularly important given the very long transient phases during which CA- and HA-MRSA can coexist. These long transient phases provide the opportunity for evolutionary adaptation of the inferior strain (by way of compensatory mutations or extension of the resistance spectrum), which could allow it to persist, even though coexistence is not expected on the basis of the current pathogen fitness.

The classical ecological paradigm of niche overlap states that two species can coexist if their resource usage differs sufficiently [33]. The present study represents an application of those concepts to the important public health question of whether hospital- and community-associated strains of MRSA are expected to coexist in the long-term. An eventual replacement of HA-MRSA by CA-MRSA could cause important changes in the epidemiology of *S. aureus*. CA-MRSA can more readily cause infections in healthy individuals than HA-MRSA [18] and hence symptomatic MRSA infections could extend to a broader class of patients. CA-MRSA strains have also been associated with a higher virulence and invasiveness than HA-MRSA strains [34], as well as worse clinical outcomes [35], [36]. This higher virulence and invasiveness has been associated with an increased expression of several cytolytic toxins (such as PVL). However, the exact mechanisms underlying the higher virulence of CA-MRSA are still uncertain [34]. An increase in virulence is also not universal, as other studies have described better clinical outcomes associated with CA-MRSA infections [37], [38]. This may be because CA-MRSA infections are largely associated with skin and soft-tissue infections, which generally have favorable outcomes [38], [39]. In addition, CA-MRSA strains have a narrower resistance spectrum which makes it easier to provide effective treatment. Overall, while the empirical evidence is mixed, there does seem to be some indication that CA-MRSA differs from HA-MRSA with regards to virulence, the range of resistance, and transmissibility (see Table 1 in [18], and [34]). Accordingly, a replacement of HA-MRSA with CA-MRSA in hospitals would entail a change in these important properties of nosocomial MRSA infections.

More fundamentally, the transmission route of MRSA in hospitals might change. The current view is that MRSA in hospitals is mainly transmitted indirectly through short-term contaminated health-care workers [40]. This dynamic could change should the more invasive and more transmissible CA-MRSA replace HA-MRSA in hospitals. Accordingly, prevention efforts that focus currently on hand-hygiene among health-care workers could lose their effectiveness in reducing the spread of MRSA. Interestingly, our results suggest that a replacement of HA-MRSA by CA-MRSA is especially likely in those locations in which infection control in hospital is currently successful and hence transmission rates in the hospital are low. However, our results also indicate that due to the large heterogeneities characterizing human populations, coexistence between hospital- and community-associated strains of MRSA is overall a likely outcome.

## Supporting Information

Equation S1 Equations summarizing the Treatment-Structured Model.
(DOCX)

Figure S1 The treatment-structured model is derived from the basic model by subdividing each compartment according to treatment status. Specifically, we distinguish between 4 treatment classes: 1) Untreated; 2) treated with a drug that is effective against neither CA-MRSA nor HA-MRSA; 3) treated with a drug that is effective against CA-MRSA but not HA-MRSA; and 4) treated with a drug that is effective against both CA-MRSA and HA-MRSA. Upon treatment initiation with an effective drug, the infection is either cleared immediately or alternatively remains colonized (this is an approximation to the real dynamics in which the patient would clear after a given amount of time). Finally, treated individuals stop treatment at a rate that is the inverse duration of antibiotic use.
(DOCX)

Figure S2 Invasion of HA-MRSA into the MSSA equilibrium: Simulations are initiated with MSSA only and run until they reach equilibrium. Then HA-MRSA is introduced (at a frequency of 0.1%). Color indicates frequency of HA-MRSA and MSSA at a given time point (for each strain we compute its frequency among S. aureus infections in the hospital and the community and use the larger of the two values): HA-MRSA alone (light blue = HA-MRSA>5% and MSSA<5%); Coexistence (dark blue = HA-MRSA>5% and MSSA>5%); MSSA alone (red = HA-MRSA<5% and MSSA>5%).
(DOCX)

Figure S3 Example runs for the treatment- and age-structured model with *R_A_^HA,H^* = 1 and fitness cost of HA-MRSA = 0.1 (A), 0.2 (B), and 0.3 (C). The system is initiated with HA-MRSA only and CA-MRSA is introduced at time 0 (thus negative time corresponds to pre-CA-MRSA frequencies of HA-MRSA). Note in pannels B) and C), the slow approach towards equilibrium (in accordance with Figure 7). All parameters other than *R_A_^HA,H^* and the fitness cost of HA-MRSA correspond to Figure 5.
(DOCX)

Table S1 Summary of the percentage of drug usage that was effective against CA- and HA-MRSA.
(DOCX)
