# Supplementary material for: Hospital-Community Interactions Foster Coexistence between Methicillin-Resistant Strains of Staphylococcus aureus
Source: PLoS Pathog. 2013 Feb 28;9(2):e1003134. doi: 10.1371/journal.ppat.1003134 (PMC3585153; doi:10.1371/journal.ppat.1003134)
Supplement: Equation S1 — Equations summarizing the Treatment-Structured Model. (DOCX) [file ppat.1003134.s001.docx]

**EQUATION S1: (Treatment-Structured Model)**

**Community**

**Hospital**
