# Supplementary figures and images for: Hospital-Community Interactions Foster Coexistence between Methicillin-Resistant Strains of Staphylococcus aureus
Source: PLoS Pathog. 2013 Feb 28;9(2):e1003134. doi: 10.1371/journal.ppat.1003134 (PMC3585153; doi:10.1371/journal.ppat.1003134)

**FIGURE S1**


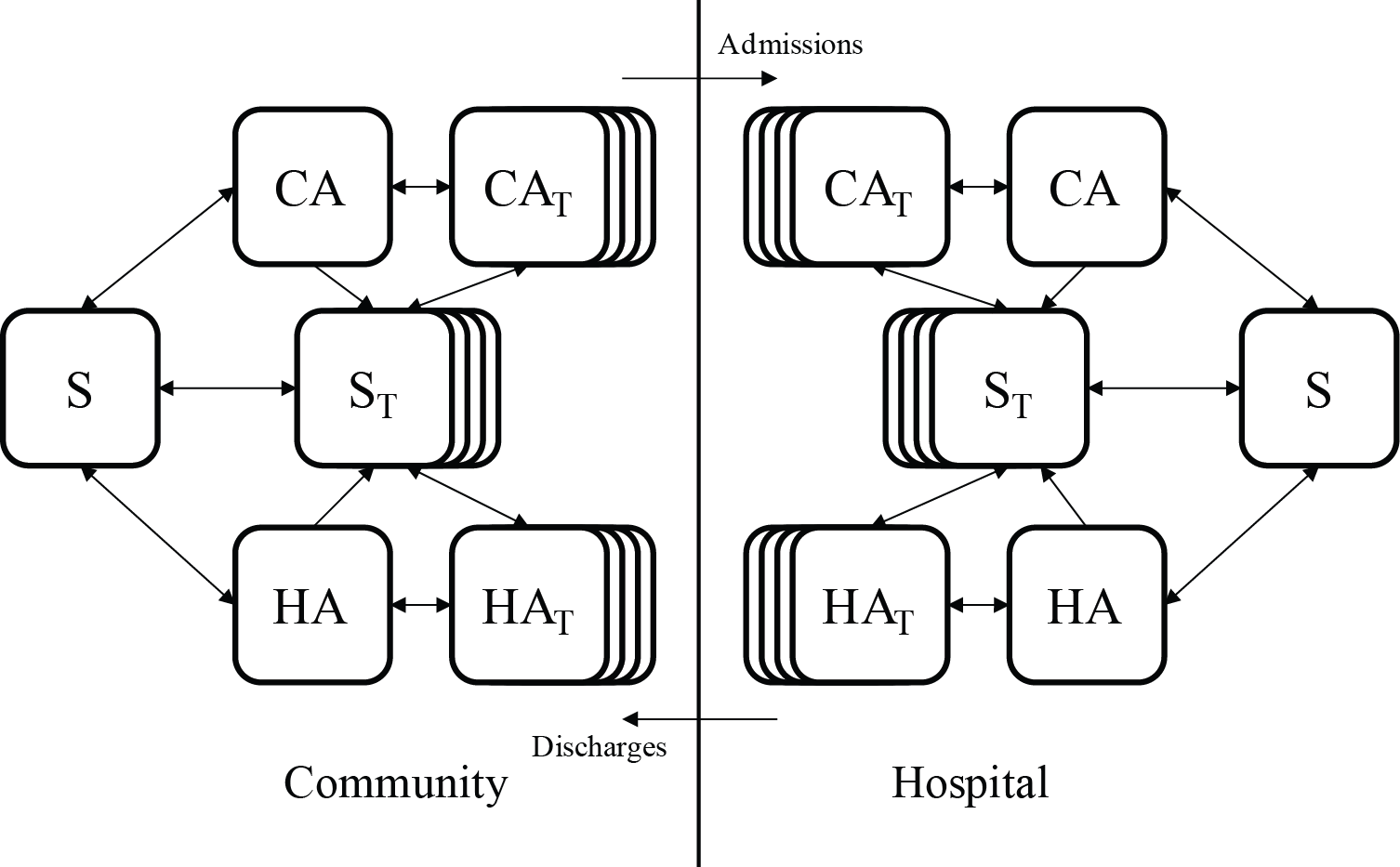

Supplement: Figure S1 — The treatment-structured model is derived from the basic model by subdividing each compartment according to treatment status. Specifically, we distinguish between 4 treatment classes: 1) Untreated; 2) treated with a drug that is effective against neither CA-MRSA nor HA-MRSA; 3) treated with a drug that is effective against CA-MRSA but not HA-MRSA; and 4) treated with a drug that is effective against both CA-MRSA and HA-MRSA. Upon treatment initiation with an effective drug, the infection is either cleared immediately or alternatively remains colonized (this is an approximation to the real dynamics in which the patient would clear after a given amount of time). Finally, treated individuals stop treatment at a rate that is the inverse duration of antibiotic use. (DOCX) [file ppat.1003134.s002.docx]
