# Supplementary material for: Hospital-Community Interactions Foster Coexistence between Methicillin-Resistant Strains of Staphylococcus aureus
Source: PLoS Pathog. 2013 Feb 28;9(2):e1003134. doi: 10.1371/journal.ppat.1003134 (PMC3585153; doi:10.1371/journal.ppat.1003134)
Supplement: Table S1 — Summary of the percentage of drug usage that was effective against CA- and HA-MRSA. (DOCX) [file ppat.1003134.s005.docx]

| **TABLE S1**  **Percentage of drug usage that was effective against CA- and HA-MRSA** | | | |
| --- | --- | --- | --- |
| **Drug Class** | **Num Prescriptions (%)** | **CA-MRSA (S/R)** | **HA-MRSA (S/R)** |
| **Aminoglycosides** | 2,955,844 (1.54%) | S | R |
| **Carbapenems** | 189,621 (0.10%) | N | N |
| **Cephalosporins** | 30,824,527 (16.04%) |  |  |
| *First generation* | 13,139,069 (6.84%) | R | R |
| *Second generation* | 5,311,534 (2.76%) | S | R |
| *Third generation* | 12,042,200 (6.26%) | S | R |
| *Fourth generation* | 205,988 (0.11%) | S | R |
| *Others* | 125,736 (0.07%) | R | R |
| **Lincomycin derivatives** | 4,854,235 (2.53%) | S | R |
| **Macrolide Derivatives** | 31,044,595 (16.15%) |  |  |
| *Ketolides* | 96,756 (0.05%) | R | R |
| *Macrolides* | 30,947,839 (16.10%) | S** | R |
| **Miscellaneous** | 14,112,618 (7.34%) |  |  |
| *Glycopeptides* | 1,293,339 (0.67%) | S | S |
| *Nitrofurans* | 5,037,964 (2.62%) | S | S |
| *Others* | 7,781,315 (4.05%) | S* | S* |
| **Penicillins** | 46,611,507 (24.25%) |  |  |
| *Aminopenicillins* | 28,307,670 (14.73%) | R | R |
| *Antipseudomonal penicillins* | 44,315 (0.02%) | R | R |
| *Beta-lactamase inhibitors* | 14,516,179 (7.55%) | R | R |
| *Natural penicillins* | 3,141,337 (1.63%) | R | R |
| *Penicillinase resistant penicillins* | 510,222 (0.27%) | R | R |
| *Other* | 91,784 (0.05%) | R | R |
| **Quniolones** | 37,438,507 (19.48%) | S | R |
| **Sulfonamides** | 14,117,926 (7.34%) | S | R |
| **Tetracyclines** | 10,064,586 (5.24%) | S | R |
| **Total** | 192,213,966 | 67% | 5% |
| * Includes antibiotics that have no activity against MRSA so does not impact resistance; ** Includes a mixture of antibiotics that are effective and not effective against CA-MRSA | | | |
